# Revisiting the earliest hyperscanning study: power and functional connectivity in the alpha band may link brains far apart

**DOI:** 10.3389/fnhum.2024.1476944

**Published:** 2024-10-08

**Authors:** Tolga Esat Özkurt

**Affiliations:** Neurosignal Laboratory, Department of Health Informatics, Graduate School of Informatics, Middle East Technical University, Ankara, Türkiye

**Keywords:** alpha activity, brain functional connectivity, coherence, data digitization, EEG, hyperscanning

## Abstract

This brief report revisits the earliest known hyperscanning study published in 1965, which examined simultaneous EEG recordings of identical twins separated by six meters. The original study’s findings suggested that eye closure in one twin elicited alpha activity in the other, despite physical separation. Leveraging contemporary signal processing techniques, we reanalyzed the digitized data to validate their findings. Spectral analysis confirmed alpha activity in the twins’ EEG signals, aligning with the original observations. Multitapering along with background noise subtraction also revealed the alpha activity in the unrelated subject, which could not be observed by visual inspection alone. Coherence analysis revealed significant alpha band synchrony between a twin and an unrelated subject, differing from the initial study’s conclusions. Our findings indicate that even historical data can yield new insights when revisited with contemporary data analysis tools and highlight the potential for future large-scale studies using advanced techniques to explore nonlocal brain interactions.

## 1 Introduction

Most human cognitive neuroimaging studies focus on individual neural activities. These studies have traditionally linked neural activities to individual cognitive and behavioral states, in addition to more recent spontaneous resting-state studies that do not involve specific experimental stimuli or tasks. However, it is reasonable to assume that, as social animals, human beings think, feel and act based on concrete or abstract social contexts. Even the presence of another person is sufficient to considerably alter resting-state neural activity, specifically EEG alpha band activity which varies with eye-contact and interpersonal distance (Gale et al., 1975). Since then, similar neural oscillatory activity changes based on the presence of another person have been reported over the years (e.g., Rolison et al., 2020).

The notion of hyperscanning, initially used by Montague et al. (2002), refers to simultaneous brain recordings (usually fMRI, fNIRS, EEG) of two or more subjects to investigate how multiple neural activities covary during various social interactions such as verbal conversations (Kawasaki et al., 2013), cooperation-competition during games (Cui et al., 2012) and simply eye-contact (Koul et al., 2023). The recent rapid rise of hyperscanning research has fostered studies linking social interaction in diverse contexts to interbrain synchrony (Müller et al., 2022).

The earliest hyperscanning report, based on the current knowledge, was authored by Duane and Behrendt (1965) and published in *Science*. This letter will focus on a reinterpretation of this pioneering study, which will be abbreviated as DB throughout the text. DB measured simultaneous EEG recordings over an occipital channel for 15 pairs of identical twins, who were seated in separate lighted rooms six meters apart. EEG data of some “unrelated” subjects were also simultaneously recorded with the twins to serve as control cases. Subjects were instructed to remain relaxed with their eyes open unless commanded to close them for a period of 5 s to 30 s. In a brief subsequent response to their critiques (Duane and Behrendt, 1966), they stated that neither the subjects nor the technicians knew the purpose of the experiment, and the command for eye-closure was given either through a whisper or a tap on the shoulder. The authors further noted that the subjects were closely monitored to ensure compliance with the commands. The authors concluded that specifically for two pairs of twins out of all, eye-closure of one consistently elicited alpha activity not only for him/herself but also for his/her twin sibling despite the latter keeping their eyes open. This was indeed a controversial and unusual result according to the cognitive psychological scientific paradigm of both then and now.

Their conclusion was said to be drawn based on gross visual inspection of pairs of occipital channel time-series. The authors provided prints of four time-series (two subplots) within the sole figure of the article. The first subplot included two temporally aligned time-series showing how eye-closure of one twin produces alpha activities in both. As an opposing exemplary case, the second subplot exhibited time-series with eye-closed alpha-activity of a twin along with those of an unrelated subject apparently with no visible alpha activity. The latter control case was yielded to illustrate how the mutual emergence of alpha activity was inexistent, when unrelated subjects were taken into account instead.

At the publication time of the aforementioned controversial but intriguing article, modern spectral and connectivity analysis methods were yet immature. In this brief letter, we digitized their data published on the article and performed contemporary signal processing methods so as to re-examine the validity of their findings. In this respect, our particular purpose was: (i) to reveal the explicit frequency content of the given data through appropriate power spectrum estimation techniques and (ii) to quantify the possible temporal co-dependence of the mutual signals using functional connectivity measures.

## 2 Methods

### 2.1 Digitization of data

For data digitization, we followed a procedure similar to that of Dalal et al. (2013), which reanalyzed intracranial electrophysiological data for cerebellar activity recorded by some studies prior to the 1960s.

The data length was 30 s. Signal traces were manually digitized from the pdf-formatted article (DB) using Engauge Digitizer,^1^ a free software tool designed for converting graphical information into numerical data. The number of samples was close to 3,000 for each time-series. To ensure exact equality of lengths of 3,000 for all four signals, a linear interpolation was applied on them using the MATLAB function *interp1*. Thus, the sampling frequency was 100 Hz for the digitized data, employed for the subsequent analyses. Please note that unavoidable data distortion due to digitization and interpolation is expected mostly to be at higher frequencies close to the Nyquist frequency of 50 Hz.

**Figure 1:**
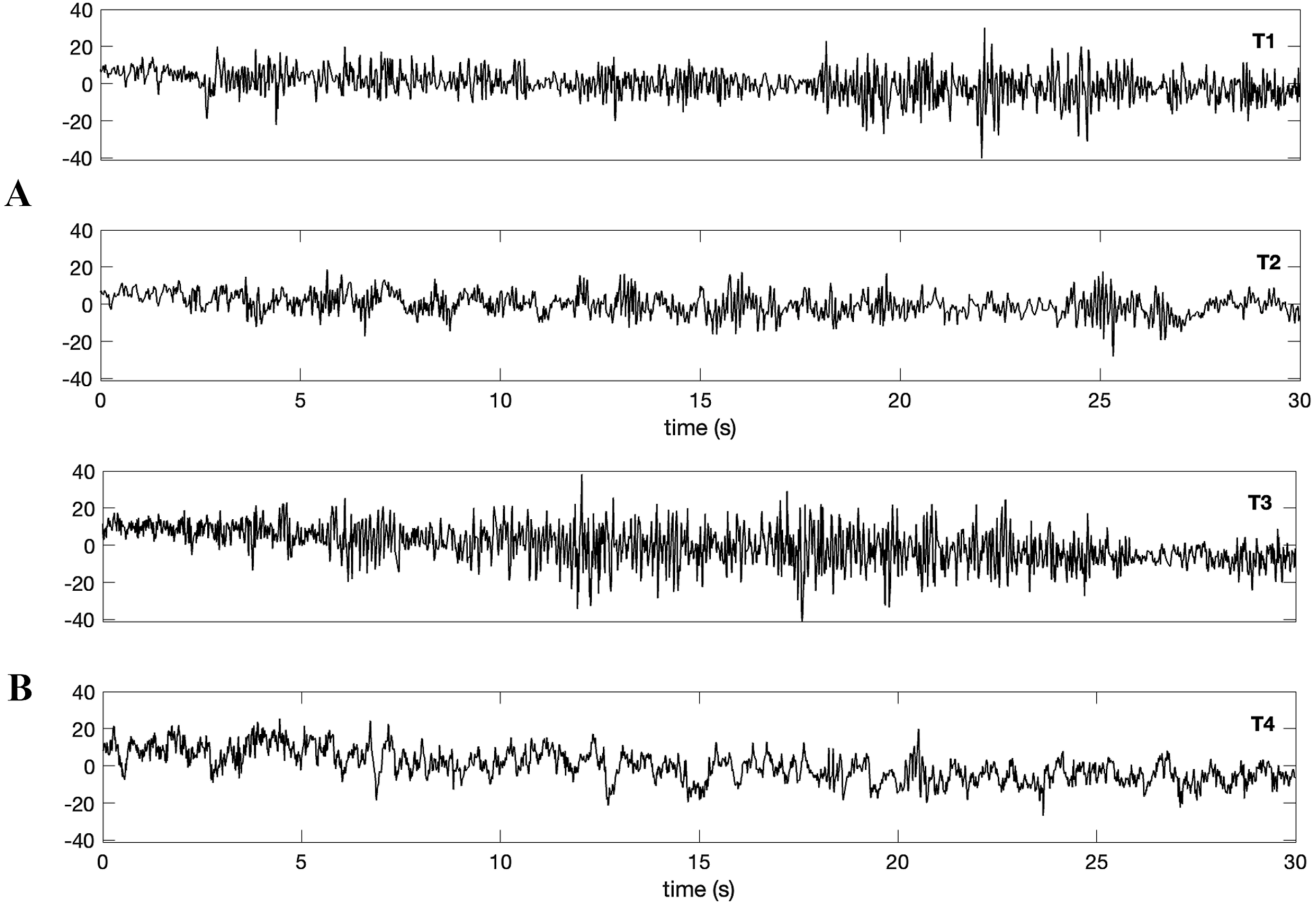
Waveforms were digitized from Duane and Behrendt (1965) as in the same order provided by the original article. Simultaneous nonlocal recordings of (A) a pair of identical twins (bottom: eye-closed T1; top: eyes-open T2) and (B) a twin and an unrelated subject (top: eye-closed T3; bottom: eyes-open T4). The data length amounts to 30 s, and the sampling rate is 100 Hz for the digitized data. Authors claimed that alpha wave emergence for the identical twin (A, top; T3), while no such induction was claimed to happen for the unrelated subject (B, bottom; T4).

Comparing the digitized EEG signals depicted in Figure 1A (supposed alpha coincidence of twins; time-series referred to as T1 and T2) and Figure 1B (supposed alpha noncoincidence between a twin and an unrelated subject by DB; time-series referred to as T3 and T4) shows that they visually resemble the original analogue recordings provided by DB.

### 2.2 Spectral and connectivity analysis

Power spectra were estimated using both Welch’s averaged periodogram method (Welch, 1967) and the multitapering method (Thomson, 1982) with half-overlapped segments of 1 s using the MATLAB functions *pwelch* and *pmtm*. For the former case, Hanning window was used to weight the segments.

Magnitude squared coherence between signals was estimated to reveal possible linear relationships between time series given in Figure 1. To this end, auto-spectra and cross-spectra were computed using the multitapering method. This particular method was deliberately selected for statistically reliable coherence estimates from relatively short signals. Data were divided into half-overlapped segments with a duration of 1 s. The number of tapers was chosen as 3, corresponding to a spectral frequency resolution bandwidth of 2 Hz. The custom software routine written for coherence analysis exploited the MATLAB function *dpss* in order to generate the tapers from Slepian sequences. In order to compute confidence limit of the coherence for each frequency nonparametrically, segments of one of the signals were randomly shuffled 1,000 times. The surrogate coherence estimate was computed between each realized shuffled signal and the intact signal. Accordingly, the confidence limit for *p* = 0.05 was obtained by taking the 95th percentile of the resultant distribution.

For amplitude–amplitude correlation of two spontaneous EEG signals (similar to the methodology given by Arnulfo et al., 2015), the following steps were performed: (i) Data were segmented into 1-s lengths with an overlap of 0.9 s. (ii) Segments were bandpass filtered (two-way least-squares FIR) using the MATLAB routine *eegfilt* of EEGLAB toolbox (Delorme and Makeig, 2004). Filter order was three cycles of the corresponding frequency band. The frequency range for filtering was 2–38 Hz, with a bandwidth of 4 Hz for each bandpass filter. (iii) Amplitudes were extracted from the absolute value of the Hilbert-transformed filtered segments. (iv) Scaled amplitude time series obtained via z-transform were inner-producted for each segment, and the correlations for all segments were eventually averaged.

## 3 Results

**Figure 2:**
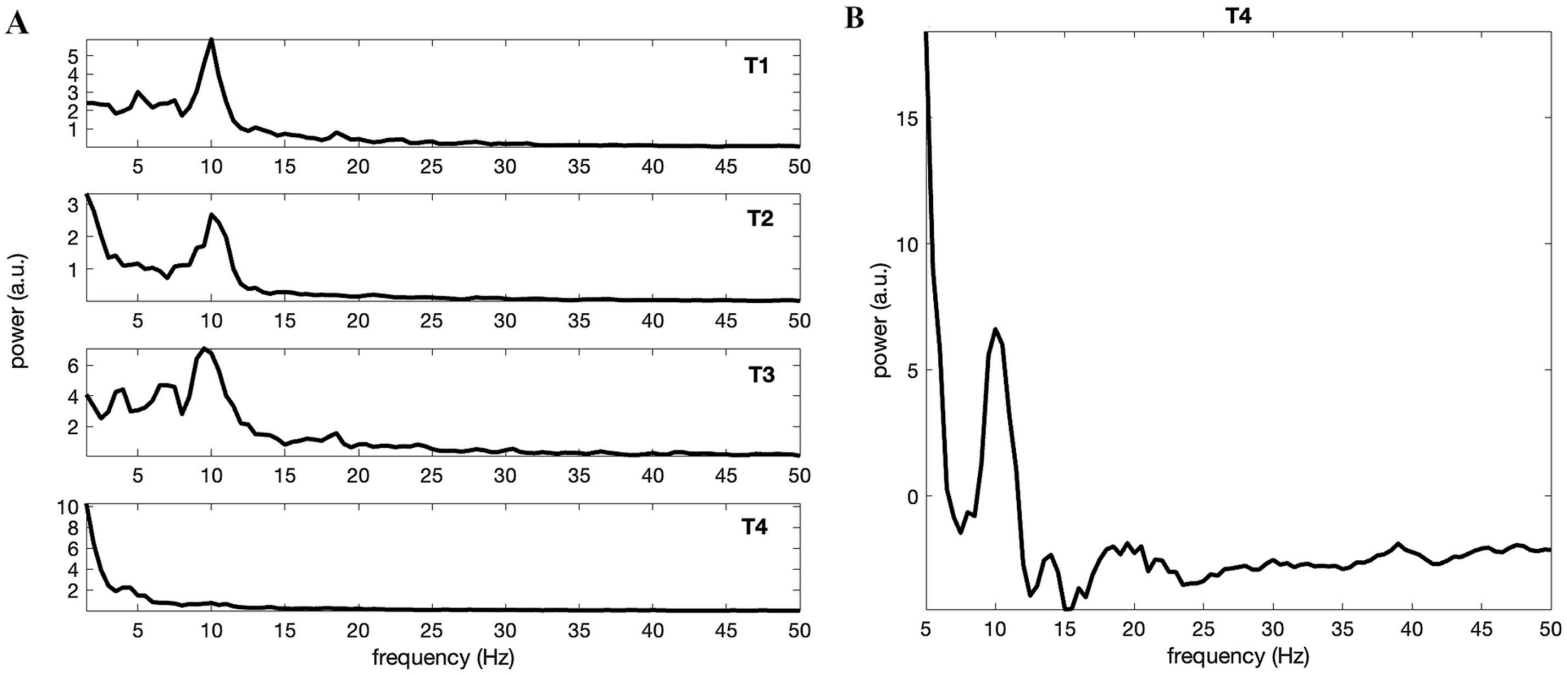
(A) Power spectra of the waveforms obtained by Welch’s method and given by Figure 1 in the same order. All but T4 possessed considerable alpha band activity peaked at about 10 Hz. This was in accordance with Duane and Behrendt (1965). (B) Spectral estimation via multitapering and 1/f component (background noise) subtraction reveal clear alpha activity also for the eyes-open unrelated subject T4.

Figure 2A depicts the power spectra (Welch’s method) of four time-series, i.e., T1, T2, T3 and T4. The spectra confirm the existence of 10 Hz alpha activity for the twins (Figure 2A). The alpha activity is not visually apparent in the time-series T4 of the unrelated subject, at least at first glance (Figure 2A, the bottom), which aligns with the findings of DB, as they noted in their figure caption: “eye closure in one of these twins” (see Figure 2A, T3) “fails to produce alpha rhythm in an unrelated subject” (see Figure 2A, T4).

Although relatively weaker in amplitude (when compared to others: T1, T2 and T3) and difficult to discern visually from the raw times series (as DB remarked) or the Welch’s method estimated spectrum alone (Figure 2A, T4), certain amount of alpha band activity of the unrelated subject becomes conspicuous when the regressed 1/f component (presumed background EEG) is subtracted from the multitapered spectrum (Figure 2B). Please see Supplementary Figure S1 depicting all spectra (T1 – T4) when multitapering and background noise subtraction were used.

**Figure 3:**
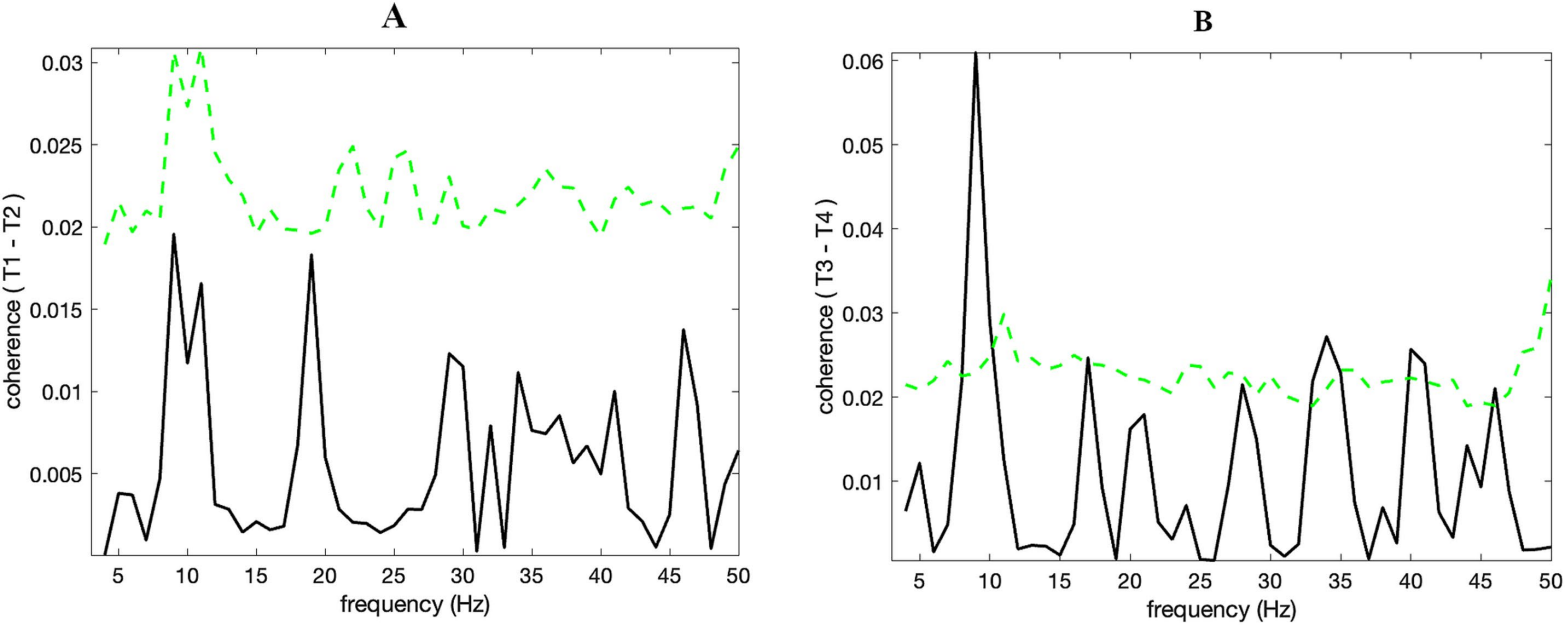
(B) There is significant (*p* < 0.01) alpha band coherence for T3 and T4, while this could not be identified so for the identical twins T1 and T2 (A). Dashed green lines show the confidence limits (*p* = 0.05) obtained through a nonparametric bootstrap from 1,000 random samples.

Despite clear alpha activities in the twins (T1, T2), no significant coherence could be identified between them (Figure 3A). In other words, there is no apparent synchrony of oscillatory activities between twins, according to the analysis. Conversely, a significant linear relationship in the alpha band was found between the time-series of a twin (T3) and the unrelated subject (T4; Figure 3B). This was rather an unexpected result as the DB had presented these time-series as an example of alpha “noncoincidence”.

**Figure 4:**
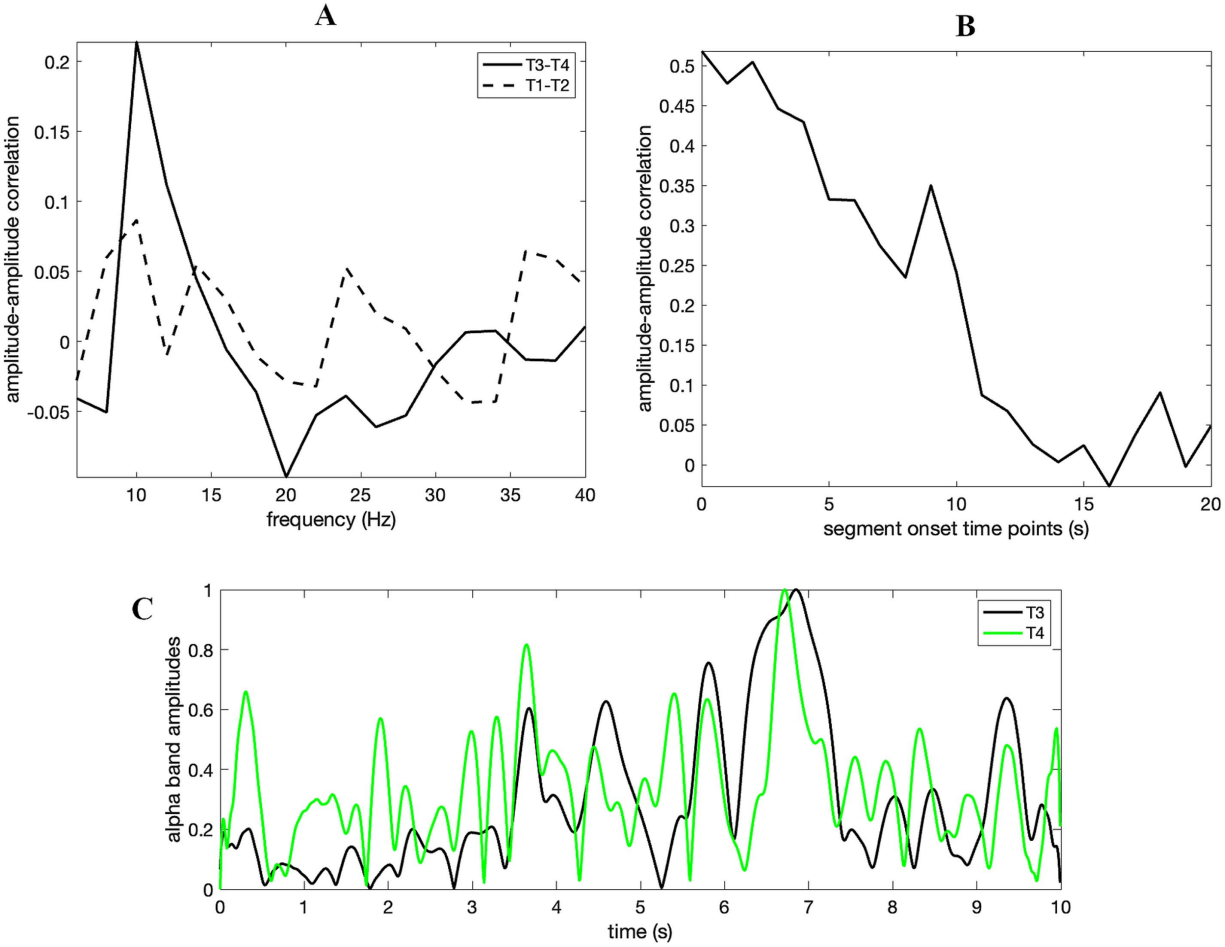
(A) Amplitude – amplitude (envelopes) correlation (obtained through its average for 10 s segments with 90% overlap) at the alpha band range between T3 and T4 (solid line) was higher than that between T1 and T2, i.e., waveforms of identical twins (dashed line). The coherence depicted in Figure 3 is produced by intersubject alpha band amplitude covariation. (B) Alpha band amplitude correlation (T3 and T4) tends to decrease monotonically through segments. Time-points in x-axis refer to the onsets of the 90% overlapped segments with 10 s length. Thus, the last point (20 s) corresponds to the onset of the last segment encapsulating the last 10 s of the data. (C) Simultaneous (each linearly scaled between 0 and 1) alpha amplitudes of T3 (black) and T4 (green) for the first segment (i.e., the initial 10 s of time-series) show how they fluctuate in tandem through time.

Additional to coherence analysis, we checked whether the alpha activity amplitude variations coincided between T3 and T4. Amplitude – amplitude correlation in the alpha band was evident for T3 – T4 and was considerably stronger than T1 – T2 (Figure 4A). This result confirmed that the significant coherence (*p* < 0.01) identified for T3 – T4 (see Figure 3B) was due to the coincidence of alpha amplitudes over time. Alpha band (8–12 Hz) synchrony gradually decreased in a nearly consistent manner as time progressed, i.e., from the first segment to the last (Figure 4B). Linearly scaled alpha band amplitudes of T3 – T4 for the first 10 s (the first segment) were overlaid in Figure 4C, in order to exhibit how the wave amplitudes of the twin and the unrelated subject coevolved in tandem. Supplementary Figure S2 exhibits the diminished correlations of the envelopes towards the middle and end of the data.

## 4 Discussion

Following the footsteps of DB, a physically isolated identical-twin functional connectivity study was recently conducted by Silberstein and Bigelow (2024). When one twin was exposed to stimuli of various images, changes in the topological connectivity of the other twin were analyzed. The authors reported a significant increase in the evoked potential event-related partial coherence between EEG channel pairs, suggesting neural interaction between twins located in separate rooms. Another recent study by Koul et al. (2023) demonstrated spontaneous interbrain connectivity when two subjects sat to face and looked at each other in the same room. As far as we know, the current brief report is the first to determine the functional connectivity of resting-state spontaneous neural activity in physically separated subjects.

Spectral analysis of the digitized data confirmed the alpha activity reported by DB for the time-series of twins (eyes-closed T2 & T3 and eyes-open T1). Additionally, although the alpha band activity in the time-series T4 (eyes-open unrelated subject) was not obvious through initial inspection, it became apparent with refined signal analysis. This outcome is expected, as resting-state occipital EEG data of human adults typically possess alpha activity even when their eyes are open, although it is amplified when eyes are closed. It should be noted that functional relevance of a neural oscillation is not just about the amplitude as such but more importantly, how it varies temporally, i.e., its dynamics.

Notably, our coherence analysis of the digitized data revealed significant synchrony in the alpha band between the eyes-closed twin (T3) and the eyes-open unrelated subject (T4). Further connectivity analysis disclosed that the synchrony originated from correlated alpha band amplitudes. Amplitude – amplitude correlation between T3 and T4 tended to decrease monotonically over time. Thus, the alpha band synchrony was highest when the eye-closure command was initiated, and the interaction diminished gradually thereafter. On the other hand, no significant coherence could be identified between signals of twins (T1 & T2). This finding was unexpected as interbrain coherence was anticipated to be stronger for the twins compared to the unrelated subjects, according to DB’s interpretation.

It is worth highlighting the difference in analysis approaches between DB and the current study to avoid potential misunderstandings. DB assessed the induction of alpha activity by visually comparing the receiver twin’s time-series during the sender twin’s eye-closure and eye-opening periods. In contrast, our study focused on the receiver’s time-series only when the sender’s eyes were closed, as this was the sole set of data available in the printed article. Therefore, our findings do not refute theirs but rather provide additional insights.

A further investigation (Behrendt and Duane, 1969) was performed by the authors of DB, this time with a different cohort comprising twins and unrelated subjects along with systematic statistical analyses. The report of the follow-up study was prepared for the US Office of Naval Research and remained publicly unknown until being made accessible recently. The experimental procedure was essentially identical to the original study DB, except some extra cautious add-ons such as the presentation of totally silent commands for eye-closure. In the report, they executed statistical analyses to capture the simultaneous presence of alpha activity beyond chance level and concluded that the coincidence of alpha waves could successfully be identified not only for the twins but also for the “unrelated” subjects. In this case, their updated conclusion can be well considered to be in line with the revealed interbrain interaction (T3 & T4) through reanalysis carried out by the current study. Please note that in their report (Behrendt and Duane, 1969), presence of “alpha coincidence” was identified based on alpha-band amplitudes. Therefore, when the amplitude within the alpha-band frequency range was weak, it was considered “non-existent.” In contrast, our use of spectral and functional connectivity analyses in the current study suggests that alpha activity with relatively weak amplitude may still play a significant functional role when nonlocal coupling is considered.

Our study generally supported the main findings of DB and further presented supplementary findings derived from significant yet limited data acquired approximately 60 years ago, made possible by advancements in signal processing throughout the years. It aims to motivate future large-scale studies involving numerous subjects and employing multichannel electrophysiological data, particularly magnetoencephalography (MEG), which is capable of capturing signals with high quality. The application of modern advanced signal processing techniques, including graph-theory network analysis, is anticipated to be crucial in resolving and elucidating the contentious issue of nonlocal brain interactions.

## Data Availability

The datasets presented in this study can be found in online repositories. The names of the repository/repositories and accession number(s) can be found at: https://github.com/tolgaozkurt/twineegspecanalysis.

## Footnotes

^1^https://markummitchell.github.io/engauge-digitizer
