# Supplementary material for: Revisiting the earliest hyperscanning study: power and functional connectivity in the alpha band may link brains far apart
Source: Front Hum Neurosci. 2024 Oct 8;18:1476944. doi: 10.3389/fnhum.2024.1476944 (PMC11493628; doi:10.3389/fnhum.2024.1476944)
Supplement: Supplementary file 1 [file Data_Sheet_1.PDF]

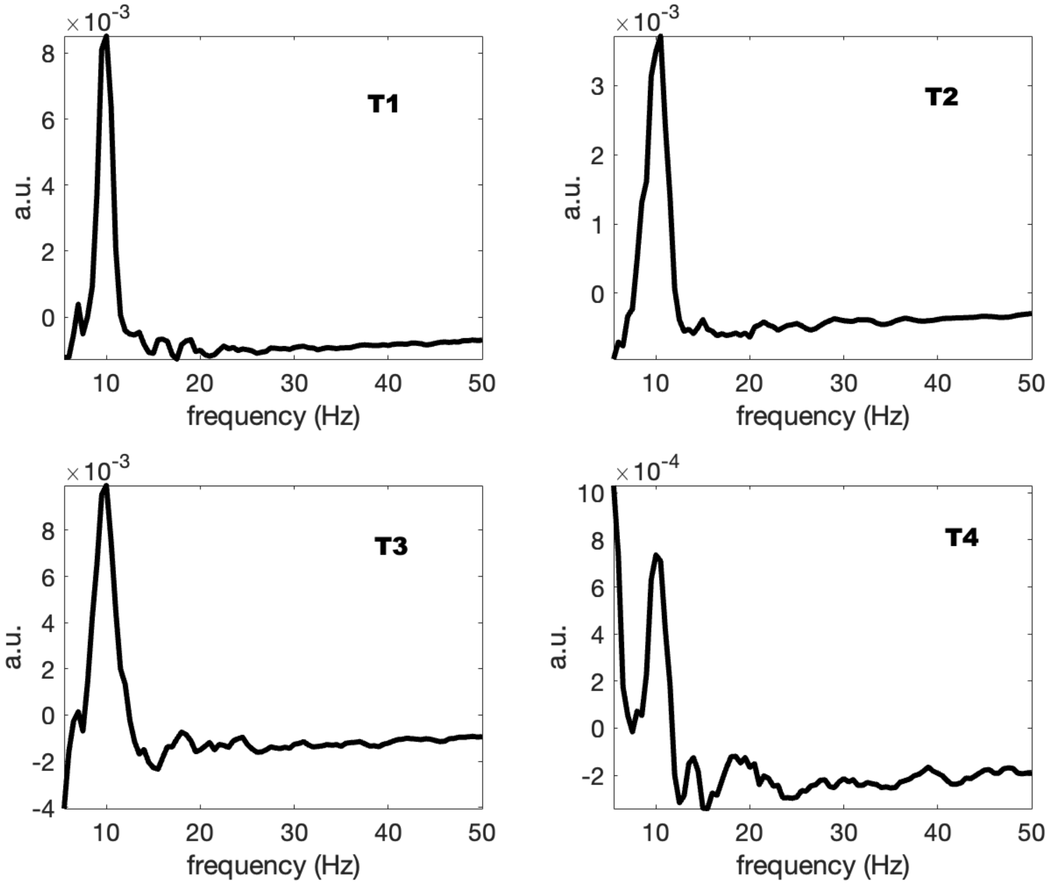

Figure S1. Power spectra of the waveforms (T1 – T4) obtained when multitapering is applied and 1/f background noise is subtracted.

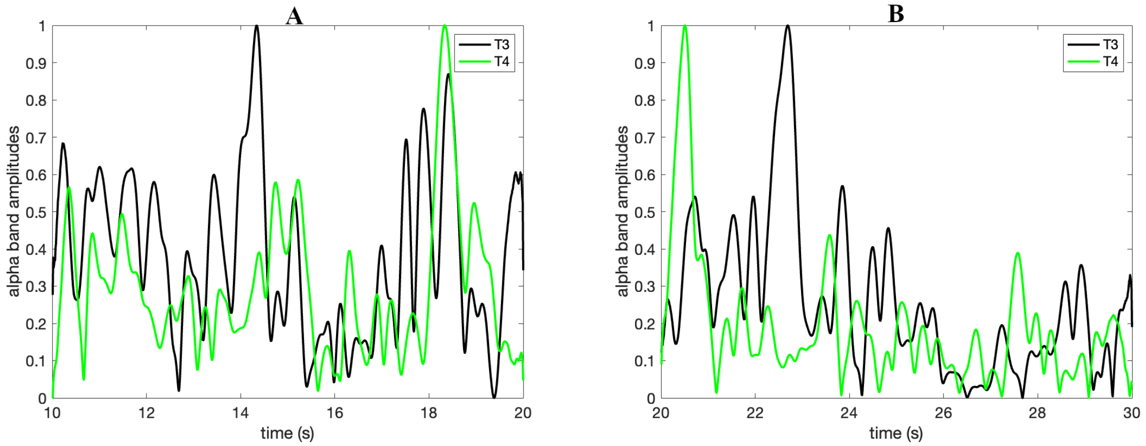

Figure S2. Alpha band amplitude – amplitude correlations (T3-T4) for the segments covering 10 – 20 s (A) and 20 – 30 s (B). The correlation is moderate for the former (A), while it is low for the latter (B).
